# Electrospun Lithium Porous Nanosorbent Fibers for Enhanced Lithium Adsorption and Sustainable Applications

**DOI:** 10.1021/acsami.4c13253

**Published:** 2024-09-30

**Authors:** Yanan Pan, Yue Zhang, Connor Thompson, Guoliang Liu, Wencai Zhang

**Affiliations:** † Department of Mining and Minerals Engineering, Virginia Polytechnic Institute and State University, Blacksburg, Virginia 24061, United States; ‡ Department of Chemistry, Virginia Polytechnic Institute and State University, Blacksburg, Virginia 24061, United States; § Department of Chemical Engineering, Department of Materials Science and Engineering, and Macromolecules Innovation Institute, Virginia Tech, Blacksburg, Virginia 24061, United States

**Keywords:** porous nanosorbent fiber, polyacrylonitrile, layered double hydroxide, DFT calculation, lithium extraction

## Abstract

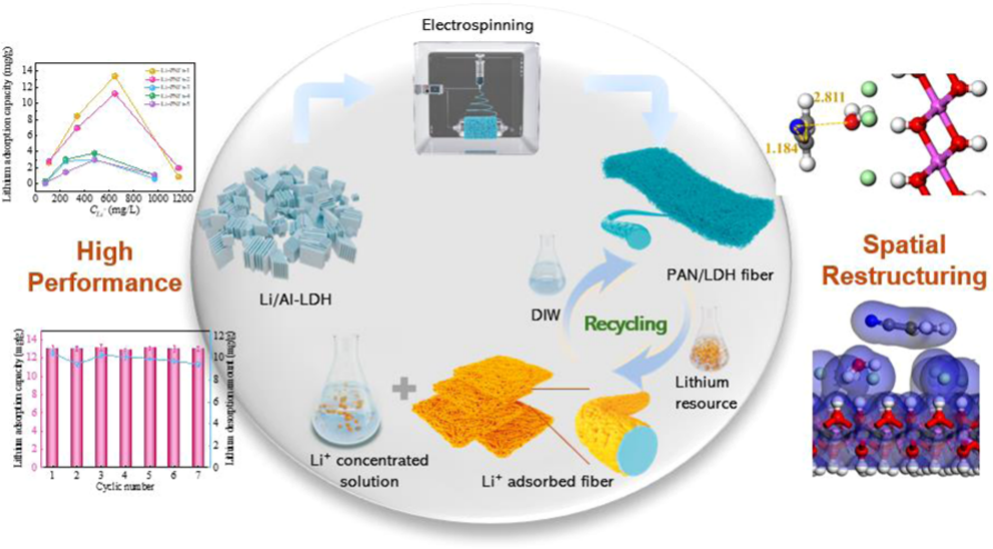

Electrospun nanosorbent fibers specifically designed for efficient lithium extraction were developed, exhibiting superior physicochemical properties. These fibers were fabricated using a polyacrylonitrile/dimethylformamide matrix, with viscosity and dynamic mechanical analysis showing that optimal interactions were achieved at lower contents of layered double hydroxide. This meticulous adjustment in formulation led to the creation of lithium porous nanosorbent fibers (Li-PNFs-1). Li-PNFs-1 exhibited outstanding mechanical attributes, including a yield stress of 0.09 MPa, a tensile strength of 2.48 MPa, and an elongation at a break of 19.7%. Additionally, they demonstrated pronounced hydrophilicity and hierarchical porous architecture, which greatly favor rapid wetting kinetics and lithium adsorption. Morphologically, they exhibited uniform smoothness with a diameter averaging 546 nm, indicative of orderly crystalline growth and a dense molecular arrangement. X-ray photoelectron spectroscopy and density functional theory using Cambridge Serial Total Energy Package revealed modifications in the spatial and electronic configurations of polyacrylonitrile due to hydrogen bonding, facilitating lithium adsorption capacity up to 13.45 mg/g under optimal conditions. Besides, kinetics and isotherm showed rapid equilibrium within 60 min and confirmed the chemical and selective nature of Li^+^ uptake. These fibers demonstrated consistent adsorption performance across multiple cycles, highlighting their potential for sustainable use in industrial applications.

## 1 Introduction

Lithium, as the key cathode material in lithium-ion batteries, plays a crucial role in shifting industries away from fossil fuels toward more sustainable energy sources.^1−3^ According to projections by the U.S. Geological Survey (USGS), global lithium consumption is expected to reach approximately 180,000 t by 2023, highlighting the rapidly increasing demand.^4,5^ Global lithium production will need to increase by approximately 500% to meet the projected energy storage demands by 2050.^6^ This surge underscores the critical need for developing more effective lithium extraction methods to meet the growing requirements for clean energy technologies. Lithium primarily occurs in various natural forms, such as lithium-bearing pegmatite deposits, Salt Lake brine reservoirs, and sedimentary deposits associated with clay minerals.^7^ Among them, Salt Lake brine is especially valuable for lithium extraction due to its high concentration of dissolved lithium, making the process both economically viable and technically feasible. This characteristic places Salt Lake brine at the forefront of lithium extraction research and development efforts, highlighting its strategic importance in the industry. Currently, the principal methods for lithium extraction from brine include solar evaporation, direct lithium extraction (DLE), membrane technologies, geothermal extraction, and electrochemical methods.^8−12^ Especially, the adsorption method of DLE is noted for its simplicity, cost-effectiveness, and high selectivity, making it a focal point.

The lithium/aluminum layered double hydroxide (Li/Al-LDH) adsorbent, known for its two-dimensional structure and specific selectivity for Li^+^ ions, is widely used in lithium extraction from industrial sources.^13^ Powdered forms of Li/Al-LDH adsorbents are typically produced using various synthesis methods, including coprecipitation, hydrothermal reactions, sol–gel processes, and mechanochemical synthesis. These adsorbents, due to their high specific surface area, rapid kinetics, versatility, and customization options, are well-suited for lithium adsorption applications from brines. For instance, Zhong et al. developed a Li/Al-LDH powder adsorbent with a significant lithium adsorption capacity of 7.27 mg/g using a straightforward one-step precipitation method.^14^ This method efficiently extracted lithium from low-grade Salt Lake brine with high selectivity, demonstrating the value for the powdered Li/Al-LDH to be utilized in real extraction. On the other hand, many Li/Al-LDH granules have been produced by molding process through wrapping powder. These granules feature enhanced mechanical strength, facilitating recycling, handling, and transportation. Pan et al. developed a novel high-mixing reactor with an antisolvent extrusion granulation strategy to develop Li/Al-LDH granules with ultrahigh powder loading.^15^ These granules demonstrated an impressive adsorption capacity of 4710.12 mg/L in the treatment of low-grade brine, significantly enhancing efficiency in practical applications. Despite extensive research on the powder and granular forms of Li/Al-LDH adsorbents, studies on their fiber forms remain notably scarce in the current scientific literature. This gap presents a promising opportunity for innovation in lithium extraction technologies.

Fiber-based adsorbents are increasingly recognized as viable options for various applications, such as wastewater treatment and metal resource recovery. Their distinctive fibrous structure facilitates rapid fluid passage through their microstructure, significantly reducing diffusion distance, which is crucial for applications that demand quick filtration and purification. For instance, Wei et al. developed iron oxide nanoneedle array-decorated biochar fibers through a hydrothermal reaction.^16^ This innovation addressed the slow adsorption kinetics typical of conventional adsorbent particles, minimized the diffusion distance for arsenic, and effectively reduced arsenic levels to below 10 μg/L within 30 min using 1.5 g/L of the adsorbent. In addition, the inherent properties of fiber materials help maintain a low pressure drop during operation, which is particularly advantageous in fluid-based systems. This characteristic reduces the energy required to sustain continuous fluid flow, enhancing overall energy efficiency. By lowering operating energy consumption, fiber materials not only improve the economic feasibility of large-scale industrial operations but also contribute to the development of more environmentally friendly and sustainable technologies. Li et al. developed a new arsenic adsorbent by coating glass fiber cloth with aqueous cerium oxide. When applied to the arsenic removal process in drinking water, it was found that the lower pressure drop facilitated a more efficient arsenic removal, surpassing the World Health Organization (WHO) standards for arsenic content in potable water.^17^ Leveraging the unique properties of fibrous materials holds great potential to significantly improve lithium adsorption and recovery from brines. However, the application of fiber-based adsorbents for lithium extraction and recovery in aqueous systems remains largely underexplored. A thorough investigation of this area could drive significant advancements, paving the way for more sustainable and efficient lithium extraction technologies tailored to diverse industrial applications.

Building on previous research, this study employed electrospinning to develop nanosorbent fibers from polyacrylonitrile (PAN) and Li/Al-LDH, aimed at improving lithium adsorption efficiency. The fabrication process was meticulously controlled and refined to ensure optimal performance. Key to this optimization was the precise adjustment and evaluation of the viscosity in the composite suspension used during fiber production, ensuring optimal fiber formation and functionality. Through thoughtful dynamic mechanical analysis, the optimal LDH content was established, achieving superior LDH particle dispersion and mechanical strength. Furthermore, the study employed dynamic contact angle measurements and N_2_ adsorption–desorption experiments to evaluate the hydrophilicity and pore structure. Detailed physical and chemical properties, along with the intrinsic action mechanism of the nanosorbent fibers, were determined using electron microscopy, X-ray diffraction (XRD), Fourier transform infrared spectroscopy (FTIR), X-ray photoelectron spectroscopy (XPS) and density functional theory (DFT) simulations. Finally, lithium adsorption mechanisms were investigated to evaluate the material’s lithium extraction performance. Adsorption–desorption cyclic experiments further demonstrated the material’s suitability for industrial applications. This work not only broadens the application range of fiber-based adsorbents but also provides a blueprint for developing more efficient lithium recovery strategies.

## 2 Results and Discussion

### 2.1 Rheological Behavior of PAN/LDH Composite Suspensions

**Figure 1:**
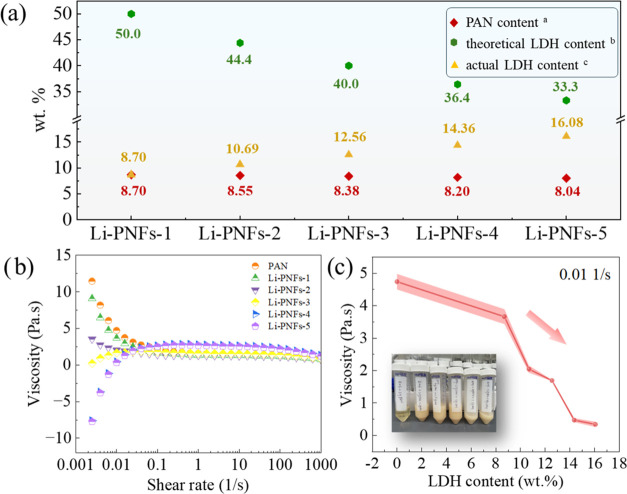
(a) Ingredient content chart; (b) Viscosity as a function of shear rate for PAN/LDH composite suspensions with different LDH contents at 293 K; (c) Viscosity of PAN/LDH composite suspensions at the shear rate of 0.01 1/s as a function of LDH content. Note: ^a^ The calculation formula of PAN content is PAN/(LDH + DMF + PAN) × 100%. ^b^ The calculation formula for theoretical LDH content is LDH/(LDH + PAN) × 100%. ^c^ The calculation formula for actual LDH content is LDH/(LDH + DMF + PAN) × 100%.

The rheological behavior of the suspensions was investigated to assess the interaction between LDH particles and the organic solution, as well as to evaluate how the mobility of the PAN/LDH composite suspensions influenced the spinning process. The PAN content and the theoretical and actual precursor LDH content of different composite suspensions are given in Figure 1a, showing the concentration of each component in the suspensions of the different fiber precursors. The viscosities of these suspensions are illustrated in Figure 1b,c. Each composite suspension exhibited non-Newtonian behavior, demonstrating pronounced shear thinning at low shear rates.^18^

Specifically, for suspensions containing 8.7 and 10.7 wt % of LDH, a sharp decline in viscosity was noted at shear rates below 0.1 1/s, attributed to the disruption of hydrogen bonds among PAN macromolecule chains.^19^ Beyond a shear rate of 0.1 1/s, the viscosities stabilized across different suspensions regardless of further increases in shear rate, indicating a flow equilibrium where the spatial arrangement and interactions within the suspensions ceased to significantly alter.^20^ Conversely, suspensions containing 12.6 wt % LDH or more showed a rise in viscosity with an increasing shear rate, gradually approaching a steady state. At shear rates under 0.1 1/s, this viscosity increment was primarily due to denser LDH particle distributions and enhanced particle interactions at higher LDH contents. As shear rates ascended, these interactions might become more pronounced, causing a tighter particle configuration and, consequently, a rise in viscosity.^21^ Eventually, the suspensions attained a flow equilibrium state.

Additionally, when the LDH content was low (≤10.7 wt %), increasing the LDH concentration resulted in a decrease in the overall viscosity. However, when the LDH content exceeded 10.7 wt %, the viscosity began to rise. This phenomenon primarily arose from the differential impact of LDH particles on the suspension structure and dynamics at varying LDH levels.^22^ At lower contents, introducing LDH particles tended to weaken particle interactions, facilitating easier dispersion and resulting in a more fluid structure. Consequently, the suspensions became more flowable, reflected by a downward shift in the viscosity profile due to reduced friction and particle interactions. On the other hand, at higher LDH contents, particle interactions became more pronounced, resulting in a denser structural arrangement. This increased particle friction, which in turn led to a rise in the viscosity profile.^23^ Furthermore, higher contents of LDH promoted particle aggregation, creating larger clusters that contributed to the increased viscosities.

The viscosity of PAN/LDH composite suspensions with varying LDH contents at a shear rate of 0.01 1/s is illustrated in Figure 1c. A shear rate of 0.01 1/s was selected as it provides an optimal balance, neither too high nor too low, making it most suitable for simulating the actual flow behavior of the suspension as it moves through the syringe during the electrospinning process. Intriguingly, an increase in LDH content led to a reduction in viscosity at this low shear rate. This trend primarily stemmed from enhanced dispersion of LDH particles within the suspension as their concentration increased. Improved dispersion lessened particle interactions, facilitating easier movement at lower shear rates and thereby decreasing viscosity. Furthermore, higher LDH contents could modify the spatial arrangement of particles within the suspensions.^24^ Additionally, at elevated LDH content levels, the shear forces might overpower particle interactions, inducing a shear thinning effect.^25^ In order to ensure better spinning results as well as better dispersion of LDH particles, low LDH content was prioritized for utilization.

### 2.2 Mechanical Strength and Hydrophilicity

**Figure 2:**
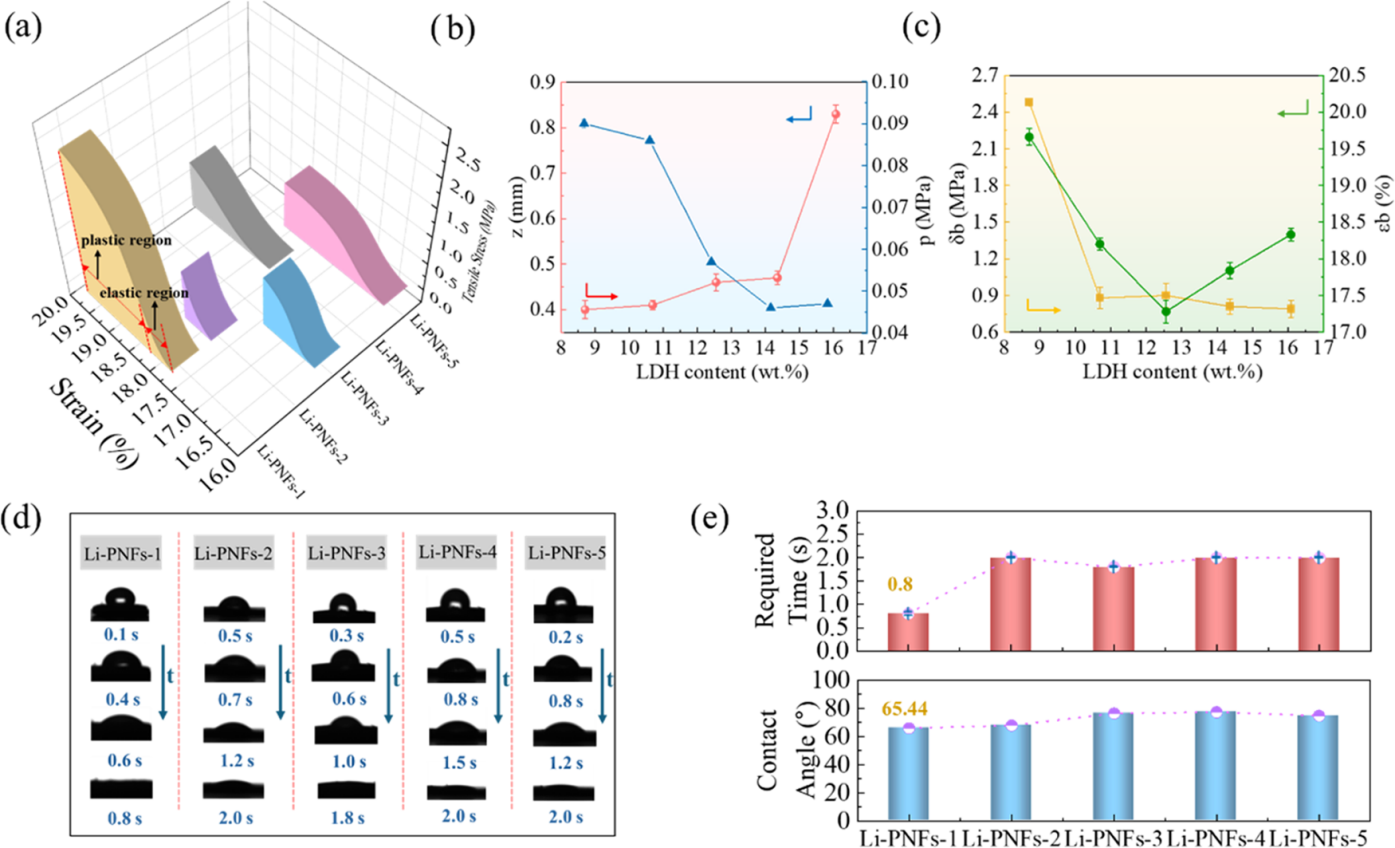
(a) Stress–strain curves of Li-PNFs; (b) Thickness *z* and yield stress *p* as a function of the LDH content; (c) Tensile strength δ_b_ and elongation ε_b_ as a function of the LDH content; (d) Dynamic waterdrop contact angles of the fiber surfaces; (e) Time required for water to completely wet the fiber surfaces and contact angle of the different fibers at 0.1 s.

Dynamic mechanical analysis (DMA) tests were conducted to evaluate the mechanical behavior of the fibers produced from the PAN/LDH composite suspensions. The resulting stress–strain curves are presented in Figure 2a. The initial gradual phase of the curves corresponds to the elastic region, demonstrating the capability to return to its original shape once the applied force is removed (consider the curve of Li-PNFs-1 as an example). Beyond the yield stress *p*, the material undergoes irreversible plastic deformation. Figure 2b displays how the *p* varies with different LDH contents. Specifically, Li-PNFs-1 demonstrated optimal shape stability with a *p* reaching 0.09 MPa. This phenomenon could be attributed to the likelihood of optimal dispersion at the low LDH content, where LDH particles were evenly distributed throughout the PAN matrix, thus offering the most reinforcement. At low contents, LDH particles could effectively dissipate the applied stresses, enhancing the overall strength and *p*. Moreover, an increase in LDH content beyond this point might lead to a saturation or reduction of the interfacial interactions, adversely affecting the mechanical performance of the composite through the poor dispersion of LDH particles.^26^ Optimal dispersion of LDH at lower contents helped minimize stress concentration points, leading to a more even stress distribution under load and consequently elevating the *p*.

The thickness *z* of the fiber flakes increased with the LDH content as depicted in Figure 2b. This phenomenon was primarily due to a larger filling volume accommodating more LDH particles. Furthermore, this trend was associated with the viscosity of the PAN/LDH suspension. As previously discussed, an increase in the LDH content resulted in a decrease in viscosity at a shear rate of 0.1 1/s, leading to thicker flake formation. A lower viscosity during the spinning process allowed the spinning fluid to flow more smoothly through the spinneret, making it more prone to forming and solidifying into thicker sheets, thereby producing thicker fibers.^27^ Additionally, a decrease in viscosity might influence the stretching and orientation of the fibers. Specifically, a lower viscosity could cause fibers to be less stretched during the process, culminating in thicker flakes after solidification and curing.^28^

Upon transitioning into the plastic region as indicated in the curve of Figure 2a, the material experienced substantial plastic deformation before arriving at the fracture point. By calculating the area under the curve, the toughness values of the materials were determined. Comparative analysis showed that the toughness ranked in a descending order as follows: Li-PNFs-1 > Li-PNFs-5 > Li-PNFs-4 > Li-PNFs-3 > Li-PNFs-2. Additionally, Figure 2c displays the tensile strength δ_*b*_ and elongation ε_*b*_ at break of the Li-PNFs, illustrating a clear trend where δ_b_ diminished as LDH content increased. Notably, at a minimal LDH content of 8.07%, Li-PNFs-1 achieved a δ_b_ of 2.48 MPa. This effect was largely attributed to the role of LDH content in facilitating the stretching and orientation of polymer chains during spinning. Optimal alignment of polymer molecular chains enhanced intermolecular forces, thus boosting the tensile strength of the fibers. Furthermore, Figure 2c indicates that ε_b_ initially decreased with an increase in LDH content under low ranges, but it began to increase as LDH content continued to rise. Achieving an ε_b_ of 19.7% when LDH content was as low as 8.07% was primarily due to improved fluidity and orientation of the polymer chains at this point, aligning with previous conclusion that δ_b_ had a large advantage at 8.07% LDH content.

The contact angles, presented in Figure 2d,e, show that the contact angle of all the fibers is less than 90° and decreases progressively as the contact time increases, confirming their hydrophilic nature. Notably, the contact angle of Li-PNFs-1 at the 0.1-s mark was 65.4°, the lowest among all the fibers tested. By comparing the hydrophilicity of the different fibers at the initial moment when the water droplet first makes contact, a representative view of their surface wetting properties could be gained. This result demonstrates that Li-PNFs-1 exhibits the highest level of hydrophilicity among the fibers analyzed. Moreover, Li-PNFs-1 reached a contact angle of 0° in just 0.8 s, demonstrating the highest hydrophilicity among the fibers tested. This characteristic is favorable for enhancing the efficiency of lithium recovery from brines since highly hydrophilic materials have better contact efficiency and lower the energy barrier of ions on the surface of the material. Overall, Li-PNFs-1 demonstrated remarkably high mechanical strength and hydrophilicity, which would facilitate Li^+^ ion recovery in solution systems while maintaining its structural integrity.

### 2.3 N_2_ Adsorption/Desorption and Morphologies

**Figure 3:**
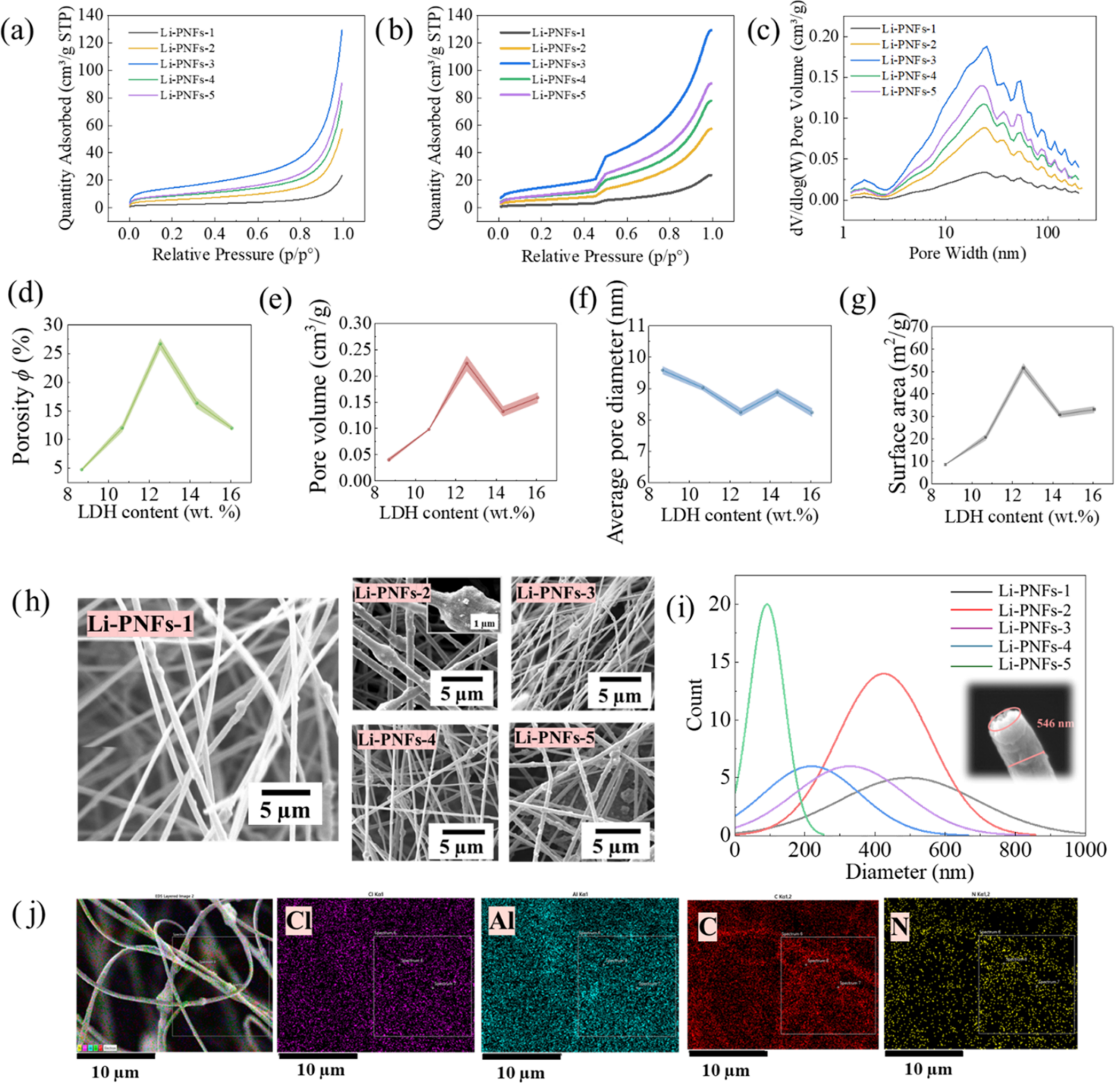
(a, b) N_2_ adsorption/desorption curves of the various Li-PNFs; (c) Pore size distributions of the various Li-PNFs; (d, e) Porosity ϕ and pore volume as a function of the LDH content; (f, g) Average pore diameter and surface area change as a function of the LDH content; (h) Morphologies of different Li-PNFs; (i) Fiber size distribution of the different Li-PNFs and high-magnification Scanning electron microcope (SEM) image of Li-PNFs-1; (j) Energy dispersive spectroscopy (EDS) elemental mapping of Li-PNFs-1.

To understand the pore structure and distribution in Li-PNFs, N_2_ adsorption/desorption experiments were conducted (Figure 3a,b). The results illustrated a gradual increase in N_2_ adsorption with increasing relative pressure, followed by a notable surge around 0.5 relative pressure. This behavior categorized the N_2_ adsorption–desorption curves of these fibers as International Union of Pure and Applied Chemistry (IUPAC) Type IV, indicative of adsorption layer formation at medium relative pressures and a hysteresis loop at the end of the curve, demonstrating capillary condensation. Furthermore, the comparative analysis of N_2_-sorption across different fibers showed a sorption capacity ranking of Li-PNFs-3 > Li-PNFs-5 > Li-PNFs-4 > Li-PNFs-2 > Li-PNFs-1, highlighting variations in pore numbers, sizes, and specific surface areas among the fibers. Figure 3c, employing nonlocal density functional theory (NLDFT), revealed the pore size distribution with a peak at approximately 1.9 nm in the micropore range and a distinct peak at 24.9 nm in the mesopore range.^29−31^

Additionally, all Li-PNFs possessed large void volumes and showed significant porosity, with the maximum porosity ϕ reaching 27.0% at a LDH content of 12.6 wt % (Figure 3d,e). Comparing the average pore sizes and surface areas among the different Li-PNFs (Figure 3f,g), all fibers exhibited an average pore size larger than 8 nm and a high surface area. Notably, Li-PNFs-3, with 12.6 wt % LDH content, demonstrated the highest specific surface area and the smallest average pore size, correlating with its superior N_2_ adsorption–desorption capacity. These Li-PNFs exhibited a layered porous structure featuring micropores, mesopores, and interfiber voids, especially Li-PNFs-3. This hierarchical porous architecture facilitated easy access of Li^+^ ions to the surface sites of the fibers, enhancing their functionality.

To compare the Li-PNFs with PAN fibers prepared under the same conditions and to elucidate the effect of LDH incorporation, BET analysis of pure PAN fibers was conducted, with results given in Figure S1. In comparison, pure PAN fibers exhibited lower N_2_ adsorption capacity, smaller specific surface area, and reduced pore volume. This is primarily due to the more complex and developed pore structure of Li-PNFs fibers. The introduction of layered LDH contributed additional micropores and mesopores, which increased the surface area and enhanced N_2_ adsorption. Moreover, the internal structure of Li-PNFs fibers was likely to be more porous and loosely packed, whereas pure PAN fibers had a relatively dense structure. Therefore, it can be concluded that Li-PNFs fibers possess a superior porous structure, providing optimal conditions for efficient lithium adsorption and capture from brine systems.

To visualize the morphology of different Li-PNFs, electron microscopic images are shown in Figure 3h. It is evident that as the LDH content increased, the surface of the originally smooth rhizomatous fibers developed increasingly porous and granular protrusions. Notably, larger and more rapid protrusions were observed in Li-PNFs-2. Moreover, with the higher LDH content, the arrangement of the fibers became more disordered. In contrast, Li-PNFs-1 exhibited a smoother and more uniform structure without disorganized protrusions, and the fibers were distributed more evenly in space. The fiber diameter analysis (Figure 3(i)) indicates that as LDH content rose, fiber diameters decreased, primarily due to the lower viscosity of the PAN/LDH composite suspension. Furthermore, the diameter of Li-PNFs-1 fibers mostly ranged between 400 and 600 nm, demonstrating superior uniformity. This uniformity not only facilitated the formation of a well-organized pore structure, enhancing the penetration and circulation of the adsorption medium, but also reduced pressure drop as fluids passed through the material, thereby improving both the efficiency and effectiveness of the adsorption process. To comprehensively examine the microscopic morphology and elemental distribution of Li-PNFs-1, high-resolution electron micrographs and energy-dispersive X-ray spectroscopy results are presented in Figure 3(i,j). The fiber diameter at the ports of Li-PNFs-1 was measured at 546 nm, featuring a hairy and uneven texture. The primary components of Li-PNFs-1 included Cl, Al, C, and N, with these elements being uniformly distributed across the analyzed regions.

### 2.4 Composition Analysis and Mechanism

**Figure 4:**
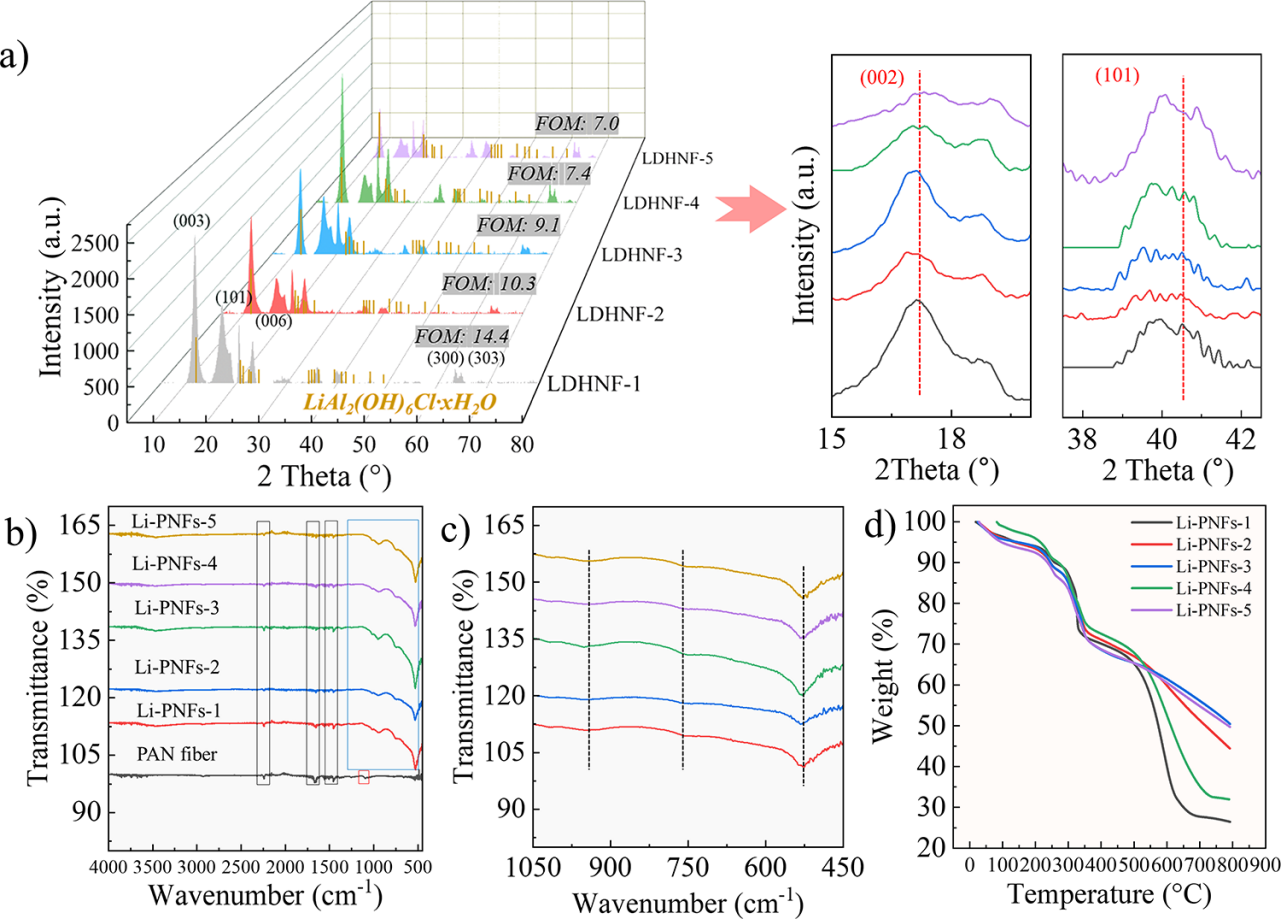
(a) XRD spectra; (b, c) FTIR spectra; (d) TGA curves of Li-PNFs.

A detailed comparison of the chemical composition and structure of different Li-PNFs is provided in Figure 4. In Figure 4a, the XRD spectra displayed crystal facets such as (003), (101), (006), (300), and (303) across all fibers, indicating the presence of Li/Al_2_(OH)_6_Cl·*x*H_2_O. Additionally, the *d*-spacing relationship of *d*_(003)_ = 2*d*_(006)_ confirmed that these new LDH-based fibers retained a regular layered structure. The figure of merit (FOM) value suggested that the crystal structure of Li-PNFs-1 closely aligned with the standard reference, pointing to a more accurately represented Li/Al_2_(OH)_6_Cl·*x*H_2_O structure, thus featuring a better theoretical lithium adsorption performance.^32^ Furthermore, the spectrum revealed two crystal facets, (002) and (101), associated with the PAN crystal structure, confirming the existence of a PAN structure in these fibers.^33^ To delve deeper into the crystal cell volume and size of the Li-PNFs, the cell parameters are presented in Table S1. Notably, the reduced cell parameters and smaller volume observed in Li-PNFs-1 could primarily be attributed to the low LDH content. This lower concentration of LDH resulted in fewer crystal nucleation sites, allowing the crystals to grow in a more orderly fashion and leading to the development of a smaller cellular structure.^34^ Additionally, the lower LDH content meant that fewer inorganic fillers were involved in the construction of the cells and the polymer molecules might be more likely to form their inherently more compact cell structure.

FTIR spectra of the fibers are presented in Figure 4b,c. The peaks at 2240, 1670, and 1458 cm^–1^ were indicative of the C≡N stretch, C=O telescoping vibration, and C–H bending vibration in PAN, respectively.^35^ Additionally, within the range of 450–1050 cm^–1^, three peaks were observed at 957, 752, and 532 cm^–1^, corresponding to the −OH vibration, Al–O oscillation, and Al–O deformation vibration, associated with the structure of Li/Al-LDH.^36^ Additionally, the atomic concentrations depicted in Figure S2 reveal that the concentrations of C and N atoms in all fibers stayed relatively stable, whereas the O atomic concentration exhibited significant variation in response to changes in LDH content. Furthermore, Li-PNFs-1 exhibited the highest concentration of O atoms. This high O atomic concentration was primarily because the low LDH content had a diminishing shielding effect over the oxygen-containing functional groups in the oxidized PAN. The greater dispersion of PAN on the surface exposed more carboxyl and ester groups introduced during the PAN oxidation process.

TGA in Figure 4d shows that the weight loss of these fibers occurred in four distinct stages. The initial stage, from room temperature to 180 °C, was primarily due to the loss of water molecules and DMF. The second stage, from 180 to 270 °C, involved the partial polymer degradation and the release of hydrogen cyanide (HCN), carbon dioxide and carbon monoxide.^37^ This was followed by the pyrolysis or oxidation of LDH interlayer ions in the temperature range 400–800 °C. The final stage concluded at 800 °C, characterized by the decomposition of components within the LDH interlayer structure and the release of other nitrogen-containing organic compounds from PAN. Detailed weight loss data of each stage for each Li-PNFs are provided in Table S2. Significantly, the fourth stage of Li-PNFs-1, which included the further decomposition of the PAN/LDH structure, exhibited the highest weight loss of 45.7%. This substantial reduction was primarily attributed to the altered decomposition kinetics of PAN/LDH with reduced LDH content and a weakened thermal stabilization effect. Consequently, more organic matter underwent decomposition.

**Figure 5:**
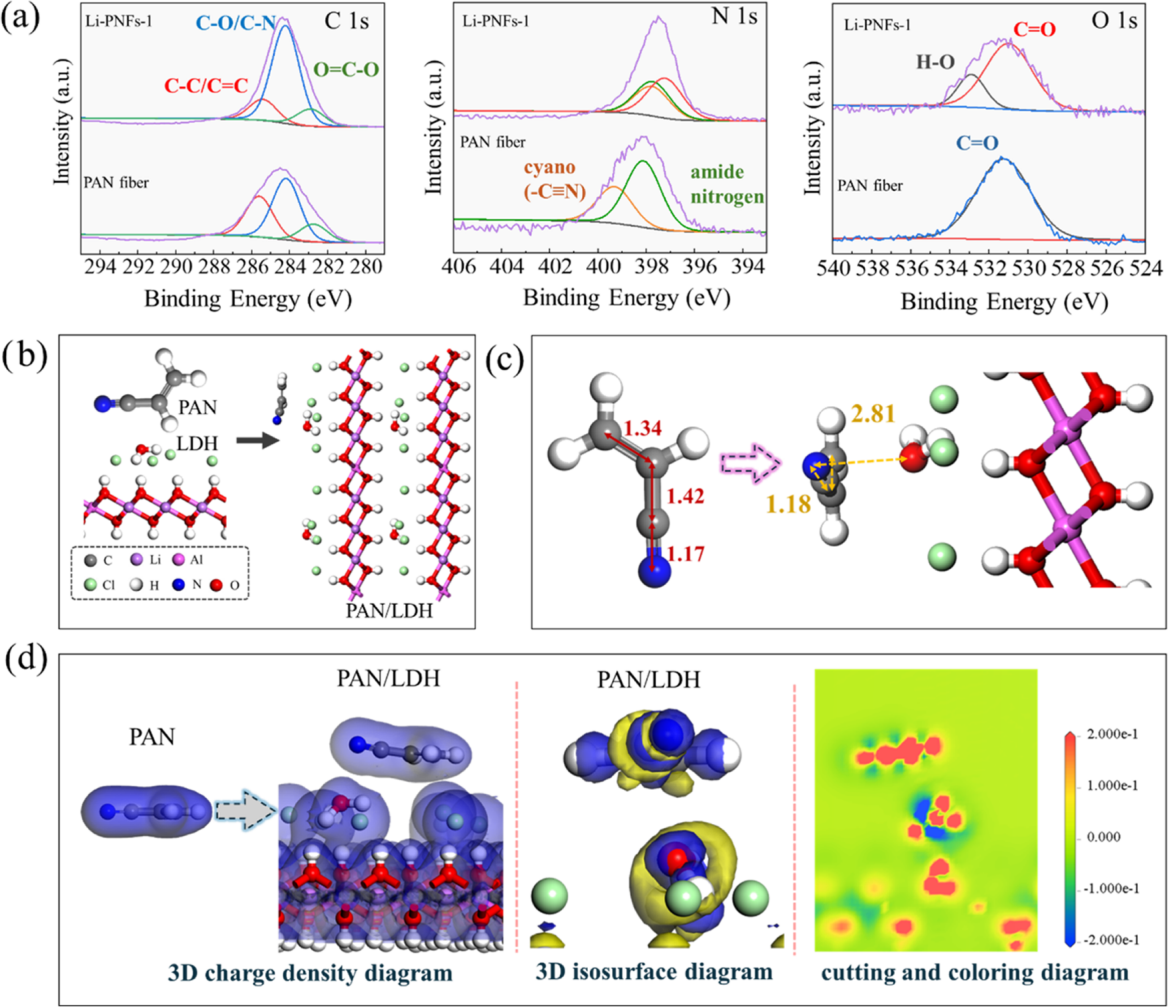
(a) XPS C 1s, N 1s, and O 1s high-resolution spectra of PAN fiber and Li-PNFs-1; (b) Structural optimization of PAN, LDH, and PAN/LDH; (c) Geometry optimization of PAN and PAN/LDH; (d) Electron density and electron difference density variation between PAN and PAN/LDH.

To clarify the mechanism of LDH interaction with PAN solution in Li-PNFs-1, Figure 5a presents high-resolution XPS spectral analysis for the chemical environments of C, N, and O atoms. Remarkably, in the PAN fiber, the peaks indicative of C–C/C=C, C–O/C–N, and O=C–O occurred at 285.40, 284.21, and 282.89 eV, respectively.^38^ For Li-PNFs-1, these peaks were slightly shifted, likely due to the interaction between the layered LDH and PAN, which altered the chemical environments of these atoms. On the other hand, peaks in the N 1s spectrum for PAN fiber, corresponding to cyano and amide nitrogen were found at 399.21 and 398.11 eV.^39^ Upon integration of the LDH structure, these peaks shifted, and a new peak emerged at 397.21 eV. This new peak’s appearance and the shifts were attributed to polar interactions: the polar –C≡N groups might form hydrogen bonds with water molecules present in the LDH interlayers, impacting the electronic environment of N atom.^40^ Another notable observation is that the –C≡N peak was slightly overlapped by the amide nitrogen peak, which can be attributed to changes in the chemical environment of the N atoms. This also affected the spatial arrangement of the PAN chain segments and their intermolecular interactions. In addition, the O 1s spectra in these two fibers were discussed. The C=O peak at 530.98 eV, corresponding to C_3_H_7_NO, remained unchanged by the introduction of LDH. However, for Li-PNFs-1, an additional peak at 532.89 eV, was assigned to the H–O, suggesting the formation of hydrogen bonds. Although individual hydrogen bonds were relatively weak, their collective effect could significantly strengthen the overall interaction, thereby improving the integrity and stability of the material. In conclusion, while the introduction of LDH would not alter the original chemical states of C, N, and O, it influenced the spatial configurations and intermolecular interactions within the fibers.

The original PAN and Li/Al-LDH models after optimization are given in Supporting Information in Figure S3. To elucidate the molecular interaction between PAN and LDH structures, the most stable configuration was identified following energy minimization, as depicted in Figure 5b. It was evident that the cyano group in PAN established a new hydrogen bond with water molecules within the LDH layer. Figure 5c illustrates the alterations in the geometric configuration before and after PAN interacting with LDH. Notably, there was a significant modification in the spatial arrangement of C, N, and O atoms, with the bond length between N and C increasing from 1.17 to 1.18 Å. Similarly, the bond lengths for C–C single and C–C triple bonds adjusted from 1.42 and 1.34 to 1.43 and 1.35 Å, respectively. The bond length for the newly formed N–O bonds was 2.81 Å. Furthermore, Table 1 presents the postoptimization energy values, indicating a binding energy of −1.95 eV between PAN and LDH, suggesting a certain degree of chemical stability in their interaction. When combined with the formation of hydrogen bonds, this chemical stability could be further enhanced by providing additional attractive forces between the polymer and the LDH surface.

**Table 1:** Calculated Energy of PAN/LDH Structure

*E*_base_	*E*_0_	*E*_tot_	*E*_ads_ (Ha)	*E*_ads_ (eV)
–24498.99	–170.66	–24669.73	–0.072	–1.95

Additionally, to assess the impact on the electrochemical environment surrounding the N atoms in PAN, variations in charge density and differential charge density are depicted in Figure 5d. This analysis showed a shift in electron density, with an increase noted in the blue regions and a decrease in the yellow regions. The charge distribution was also altered, where increased charge was represented by red areas and decreased charge by blue. In summary, the interaction between PAN and LDH resulted in a chemically stable structure facilitated by hydrogen bonding. While the original chemical states of C, N, and O atoms remained unchanged, there were notable changes in the geometrical spatial structure, intermolecular interactions, and the electrochemical environment of the N atoms.

### 2.5 Lithium Adsorption and Cyclic Stability

**Figure 6:**
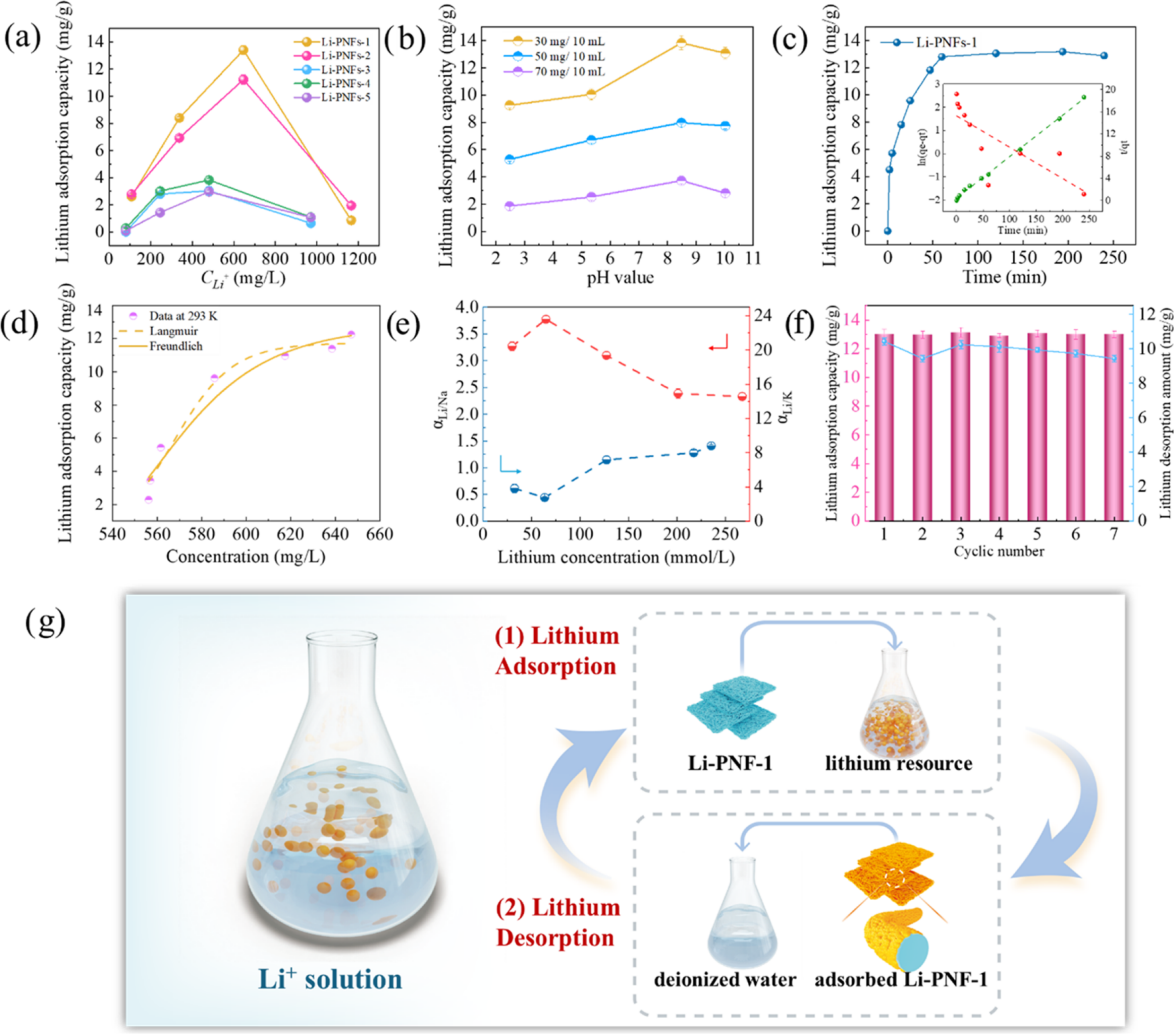
(a) Effect of initial lithium concentration on the lithium adsorption capacity of Li-PNFs; (b) Effect of pH value and fiber amount of Li-PNFs-1; (c) Lithium adsorption kinetics and fitting curves using Li-PNFs-1; (d) Lithium adsorption isotherm using Li-PNFs-1; (e) Adsorption selectivity for Li-PNFs-1; (f) Lithium adsorption and desorption cyclic performance of Li-PNFs-1; (g) Schematic diagram of the lithium extraction by Li-PNFs-1.

To assess the lithium extraction performance of Li-PNFs, lithium adsorption experiments were carried out in systems containing different initial Li^+^ concentrations (Figure 6a). The adsorption behavior of various Li-PNFs demonstrated a pattern where it first increased and then decreased with the increasing Li^+^ concentration. This pattern was primarily attributed to the enhanced driving force for lithium adsorption as the concentration of Li^+^ ions increases initially. Conversely, at higher concentrations, the decrease in adsorption was mainly due to the oversaturation of adsorption sites and detrimental changes in the chemical environment of the adsorption medium. These changes, triggered by high concentrations of Li^+^ ions, led to the desorption of previously adsorbed Li^+^ ions. Specifically, the Li-PNFs-1 exhibited the most effective lithium adsorption, achieving a maximum capacity of 13.45 mg/g at an initial Li^+^ concentration of approximately 600 mg/L. This superior performance was primarily attributed to an optimal Li/Al-LDH content within the fiber, facilitating a more uniform dispersion of adsorption sites across the fiber, thereby preventing aggregation and reduced effective surface area observed with higher LDH concentrations.

On the other hand, the influence of pH and fiber dosage is illustrated in Figure 6b, showing that a lower amount of fiber resulted in higher adsorption levels. This effect was attributed to the fact that using fewer fibers led to a higher concentration of Li^+^ ions available in the system, enhancing the driving force for adsorption.^41^ Additionally, the adsorption effect initially increased and then decreased with the rising pH, reaching an optimal level under weakly alkaline conditions. This peak in adsorption efficiency was mainly attributed to the amide group in PAN achieving its optimal protonation state in these conditions, which most effectively facilitated the interaction between PAN/LDH and Li^+^ ions.

To further elucidate the lithium adsorption mechanism, lithium adsorption isotherm and kinetics using Li-PNFs-1 are presented in Figure 6c,d. The detailed fitting parameters are given in Table S3. The lithium adsorption reached equilibrium within 60 min, stabilizing at around 13.00 mg/g. Following the adsorption process, the lithium concentration decreased by 10.45%, from 624.38 to 557.13 mg/L. Additionally, this behavior followed the pseudo-second-order model, suggesting that the adsorption process is driven by chemical reactions. The adsorption rate was justified to be influenced by the availability of adsorption sites and the formation of chemical bonds during the process.^42^ The adsorption isotherm at 293 K followed the Langmuir model, suggesting that the adsorbent’s surface contained homogeneous adsorption sites, each capable of adsorbing only one molecule.^43^ Once an adsorption site was occupied, no additional molecules could be adsorbed at that site. Moreover, the adsorption equilibrium remained constant and did not fluctuate with temperature changes. The adsorption selectivity is highlighted in Figure 6e, demonstrating Li-PNFs-1′s effective selectivity for Li^+^ and its greater selectivity for Li^+^ over K^+^ compared to Na^+^, which was primarily due to the larger ionic radius of K^+^ relative to Na^+^.

The adsorption–desorption cycling performance of materials is crucial for evaluating their suitability for recycling and industrial applications. Figure 6f illustrates the adsorption capacity when Li-PNFs-1 was subjected to seven cycles. Notably, the lithium adsorption capacity of Li-PNFs-1 remained stable at 13.00 mg/g even after seven cycles, underscoring the material’s excellent recyclability and regeneration potential. Besides, the desorption cyclic performance was also stable, remaining at 9.43 mg/g after 7 cycles. This stability provided compelling evidence of the industrial adaptability of Li-PNFs-1, showcasing its consistent performance over multiple cycles. A schematic diagram illustrating the utilization of Li-PNFs-1 in the lithium extraction process is presented in Figure 6g. During the adsorption phase, Li^+^ ions were effectively captured by the nanosorbent under specific conditions. Subsequently, the adsorbed nanosorbent underwent desorption using deionized water (DIW) to release the Li^+^ ions into the surroundings. Following this, the Li-enriched solution was reclaimed for recycling, while the regenerated Li-PNFs-1 could be reintegrated into subsequent adsorption and desorption cycles.

Table 2 provides a comparison of different adsorbents used for lithium extraction from brines. Among inorganic adsorbents, titanium-based and manganese-based adsorbents are widely studied due to their significant lithium adsorption capacity, achieving 20–40 mg/g in low-concentration brines. However, their challenges in industrial-scale application, such as high-cost and high material collapse, limit their practical utility. Alternatively, various forms of Li/Al-LDH adsorbents have been researched to different extents. Granular Li/Al-LDH adsorbents have shown promising performance in Qarhan Salt Lake brines, and powder Li/Al-LDH adsorbents have achieved a maximum lithium adsorption capacity of 13.4 mg/g. Nevertheless, fiber Li/Al-LDH adsorbents have only been explored in the work of Ding et al., with a reported adsorption capacity of just 5.2 mg/g. In comparison, the Li-PNFs-1 nanosorbent fibers prepared using electrospinning in this study demonstrate a significant improvement, with an adsorption capacity of up to around 13.00 mg/g. In conclusion, the adsorbent developed in this study fills the research gap for Li/Al-LDH in fiber form, expanding the application potential of Li/Al-LDH adsorbents and providing an efficient new method for lithium resource recovery.

**Table 2:** Summary of Different Adsorbents Employed in Lithium Adsorption from Brines and Their Performance Comparison

resource	*C*_Li_^+^ (mg/L)	adsorbent type	adsorbent	adsorption capacity	refs
oil-gas field brines	100	titanium-based adsorbent	TiO_2_·H_2_O	20.14 mg/g	Zhang et al.^44^
untreated natural brine			FP@TiO_2_ (TiO_2_ coated on the FP surface)	32.17 mg/g	Hu et al.^45^
simulate Salt Lake brine	200	manganese-based adsorbent	ZrO_2_@HMFO	35.8 mg/g	Li et al.^46^
synthetic brine	700		HMO submicrometer-sized particles	31.1 mg/g	Arrua et al.^47^
Qarhan Salt Lake brines	400	Li/Al-LDH granule	HMAG	volumetric adsorption capacity: 4710.12 mg/L	Pan et al.^15^
Qarhan Salt Lake brines	350		PSF–PEG600@AD	retention of 90% after 20 cycles	Gao et al.^48^
Luopupo brine		Li/Al-LDH powder	Zn^2+^-doped Li/Al-LDH	13.4 mg/g	Zhang et al.^49^
West Taijinar Salt Lake brines	400		Co-LDHs-SO_4_	10.74 mg/g	Huo et al.^50^
Qarhan old brine	350		HCLDHs	9.72 mg/g	Chen et al.^13^
real Salt Lake brine	1085	Li/Al-LDH fiber	Li/Al-LDHs nanofibers from Kaolin	5.2 mg/g	Ding et al.^51^
simulate Salt Lake brine	600		Li-PNFs-1 nanosorbent fibers	13.00 mg/g	this work

## 3 Conclusions

Porous nanosorbent fibers were designed for efficient lithium extraction and prepared using electrospinning. Preliminary viscosity tests and mechanical kinetic experiments demonstrated that the optimal dispersion of LDH particles within the fibers occurred at the lowest LDH content, resulting in Li-PNFs-1 with superior shape stability, toughness, and strength. Moreover, dynamic contact angle tests indicated that Li-PNFs-1 exhibited enhanced hydrophilicity. N_2_ sorption highlighted a hierarchical porous structure within these fibers, featuring both micropores and mesopores. The morphological analysis confirmed a smooth and uniform fibrous structure for Li-PNFs-1, with more compact cellular parameters suggesting a denser molecular arrangement than the rest fibers. Furthermore, the interaction between LDH and the PAN matrix triggered a reorganization of the PAN structure upon LDH integration. Besides, lithium adsorption revealed that Li-PNFs-1 achieved superior lithium adsorption, reaching equilibrium within 60 min at about 13.00 mg/g. Kinetics and isotherm indicated that Li^+^ ion adsorption by Li-PNFs-1 was chemically driven and highly selective. The fiber’s adsorption capacity remained stable across seven adsorption–desorption cycles, underscoring the significant potential of Li-PNFs-1 for industrial applications. In conclusion, this study paves the way for the future development of efficient and sustainable lithium adsorbent materials. With further optimization of fiber structure and enhancement of adsorbent performance, Li-PNFs-1 and its analogs hold significant potential for large-scale lithium recovery applications.

## 4 Experimental Section

### 4.1 Materials

LiCl (≥99 wt % purity), AlCl_3_·6H_2_O (>99 wt % purity), NaOH (≥97 wt % purity), HCl (35–38 wt %, ACS grade), PAN (≥99 wt %, AR grade), and *N*,*N*-dimethylformamide (DMF, >99.7 wt %, AR grade) were purchased from Thermo Fisher Scientific and directly used without further purification. A synthetic brine was employed to assess lithium adsorption performance. The detailed composition and raw pH value of the brine are given in Table S4. The pH levels during specific adsorption experiments were controlled using HCl and NaOH solutions.

### 4.2 Fabrication of Li/Al-LDH and Porous Nanosorbent Fibers

**Figure 7:**
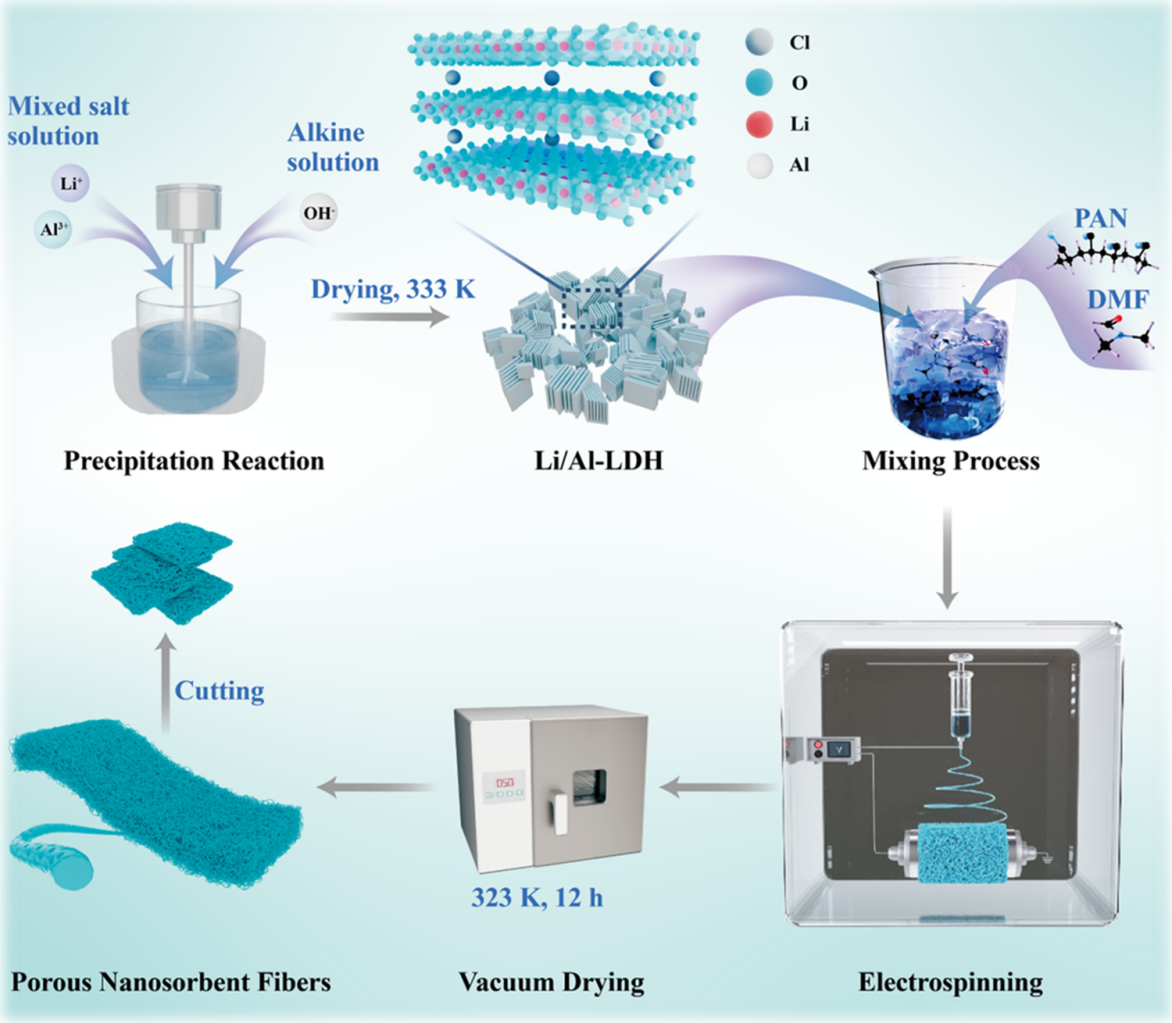
Schematic diagram of the Li-PNFs preparation procedures.

Li/Al-LDH was synthesized using a one-step coprecipitation method.^52^ As shown in Figure 7, a mixed salt solution with a Li to Al molar ratio of approximately 0.25 was gradually introduced into a base solution with a high alkaline concentration, initiating the coprecipitation reaction. The Li to Al molar ratio of 0.25 was chosen because it provides the optimal lithium adsorption and structural stability for the Li/Al-LDH product. The reaction was conducted in a jacketed glass reactor under continuous stirring. The resulting product was washed either with DIW, followed by drying at 333 K for 24 h to obtain a powder product. On the other hand, a specific quantity of PAN was dissolved in DMF at 333 K by stirring at 800 rpm for 2 h to achieve a homogeneous PAN solution. Afterward, a certain amount of Li/Al-LDH powder was introduced into the PAN/DMF solution and stirred at 1500 rpm for 1 h at 313 K to create a suspension. By employing this suspension, the risk of uneven LDH particle dispersion could be significantly reduced through a systematic mixing protocol and meticulous control of temperature and agitation parameters. This could ensure uniform LDH distribution in the resulting fibers after the spinning process, enhancing the overall quality and performance of the final material. Next, the suspension was loaded into a 10 mL syringe and positioned at the electrospinning machine. The suspension was pumped into a high-voltage electric field under controlled conditions of speed, humidity, and voltage. The resulting fibers were collected at a distance of 40 cm from the nozzle. Subsequently, the product was vacuum-dried at 323 K for 12 h. The final dried products were cut into 8 mm × 8 mm square flakes to obtain the Li/Al-LDH porous nanosorbent fibers (Li-PNFs). The specific parameters are provided in Table S5, while the visual representation of the product can be found in Figure S4. The nanosorbent fibers, prepared from suspensions with varying LDH contents, were named Li-PNFs-1, Li-PNFs-2, Li-PNFs-3, Li-PNFs-4, and Li-PNFs-5, respectively (see Table S5).

### 4.3 Characterizations

Viscosity was measured using a TA Discovery Hybrid Rheometer HR-3 with a 1000-μm gap and a shear rate ranging from 0.0001 1/s to 2000 1/s. DMA was conducted at 28 °C using a DMA Q800 analyzer. The dynamic contact angle was tested with a Theta Flow Tensiometer. Specific surface area and pore size of Li-PNFs were analyzed using N_2_ adsorption–desorption isotherms at 77 K with a Micromeritics-3Flex analyzer, employing the BET and BJH methods. The thickness *z* and edge length *d* of the various samples were measured using high precision vernier calipers (SYLVAC SWISS QUALITY). Additionally, the porosity ϕ of the fibers could be calculated using eq 1
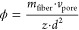
1where *m*_fiber_ (g) refers to the BET tested-fibers mass; *v*_pore_ (cm^3^/g) is the pore volume which can be obtained from BET results; *z* (cm) means the thickness of each fiber piece; *d* (cm) represents the side length of the square piece, with *d* set to 0.8 in this work.

SEM and EDS were performed to analyze morphology and elemental composition, respectively. The crystalline structure was assessed using XRD from 5 to 80° with Ni-filtered Cu–Kα radiation. FTIR analyses were conducted using a Thermo Fisher Scientific Nicolet instrument with samples prepared as KBr pellets. A Thermogravimetric Analyzer (TGA 5500, TA Instrument), featuring a high-temperature furnace, was used at a heating rate of 20 °C/min under a nitrogen atmosphere flowing at 20 mL/min. XPS analysis was carried out using an advanced PHI Quantera Hybrid system. Ion concentrations in solution were determined by inductively coupled plasma mass spectrometry (ICP-MS).

### 4.4 DFT Calculations

The DFT calculations were carried out with the Cambridge Serial Total Energy Package (CASTEP) code. The Perdew–Burke–Ernzerhof (PBE) functional within generalized gradient approximation (GGA) was used to process the exchange-correlation, while the projector augmented-wave pseudopotential (PAW) was applied with a kinetic energy cutoff of 381.0 eV, which was utilized to describe the expansion of the electronic eigenfunction.^53,54^ The Brillouin-zone integration was sampled by a Γ-centered 10 × 10 × 10 Monkhorst–Pack *k*-point. All atomic positions were fully relaxed with SCF tolerance of 1 × 10^–5^ eV. The electronic minimizer used density mixing.

The adsorption energy (eq 2) of the system was predicted from the difference between the energies of the species before and after adsorption.
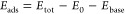
2where *E*_tot_ is the total energy of the adsorption system, *E*_0_ is the energy of the adsorbent surface, and *E*_base_ is the energy of the adsorbate.

### 4.5 Adsorption Experiments

Lithium adsorption experiments were conducted to assess the efficacy of Li-PNFs. In each experiment, a measured amount of fiber was added to a fixed volume of brine. The mixture was afterward agitated at 300 rpm for 4 h in a water-bath shaker (Series number 290400, Boekel Scientific), facilitating sample collection. Initially, the adsorption experiments were performed on brines with varying lithium concentrations, specifically 100, 300, 600, and 1000 mg/L, to determine the adsorption capability of the fiber across different lithium concentrations. Additionally, to assess the impact of pH on lithium adsorption, brines with varying pH levels were tested. Kinetic studies of the adsorption process were conducted at ambient temperature, with samples collected at specific time intervals of 0, 2, 5, 15, 25, 45, 60, 120, 200, and 240 min. Isotherm experiments related to adsorption were also carried out at ambient temperature. The lithium adsorption capacity was calculated using eq 3.
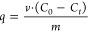
3where *C*_0_ (mg/L) and *C*_*t*_ (mg/L) refer to the ion concentration before and after adsorption, respectively; *m* (g) is the mass of the added fiber; *v* (L) is the volume of the feed brines. Details on the kinetic and isotherm fitting methods can be found in the Supporting Information.

To examine adsorption selectivity, solutions containing two metal components with a molar ratio of Me/Li = 1:1 were used, where Me denotes either Na or K. The distribution coefficient *K*_Me_ (L/g) and selectivity factor α_Li/Me_ were calculated as eq 4 and eq 5, respectively
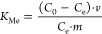
4
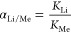
5where *C*_0_ (mg/L) and *C*_e_ (mg/L) refer to the initial ion concentration and equilibrium ion concentration, respectively; *m* (g) is the fiber mass; *v* (L) is the solution volume. The subscript *M*_e_ of *K*_Me_ and α_Li/Me_ means the metal ions in the solution.

The adsorption–desorption cycle process was conducted to assess the reusability of the nanosorbent fiber. The desorption procedure was conducted at ambient temperature. Following adsorption, the fiber loaded with lithium was vacuum-dried at 323 K for 2 h. It was treated in a fixed-bed shaker with DIW at a solid/liquid ratio of 50 mg/10 mL for 2 h under neutral conditions, resulting in regeneration of the fiber. The lithium desorption amount, *d*_e_ (mg/g), can be determined by measuring the lithium concentration in the final solution after treatment under neutral conditions, as described by eq 6
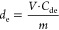
6where *C*_de_ (mg/L) means the lithium concentration in the final desorption solution; *m* (g) is the weight of used fibers; *V* (L) is the volume of used deionized water. To assess the stability of lithium adsorption performance and the fiber structural integrity, cyclic experiments involving repeated adsorption/desorption phases were conducted.

## Supporting Information Available

N_2_ adsorption/desorption curve; pore size distribution; atomic concentration; crystal cell parameters; weight loss of different phases of different fibers; DFT calculation models of PAN and LDH; lithium adsorption fitting parameters and fitting models; detailed composition of the synthetic brine; experimental parameters; photos of the real Li-PNFs fibers (PDF)
