# Supplementary material for: Electrospun Lithium Porous Nanosorbent Fibers for Enhanced Lithium Adsorption and Sustainable Applications
Source: ACS Appl Mater Interfaces. 2024 Sep 30;16(40):54260–72. doi: 10.1021/acsami.4c13253 (PMC11472274; doi:10.1021/acsami.4c13253)
Supplement: Supplementary file 1 — am4c13253_si_001.pdf [file am4c13253_si_001.pdf]

Supporting Information

Electrospun Lithium Porous Nanosorbent Fibers for Enhanced Lithium Adsorption and Sustainable Applications

Yanan Pan [a], Yue Zhang [b], Connor Thompson [b], Guoliang Liu [b,c], Wencai Zhang [a,*]

[a] *Department of Mining and Minerals Engineering, Virginia Polytechnic Institute and State University, Blacksburg, Virginia 24061, USA*

[b] *Department of Chemistry, Virginia Polytechnic Institute and State University, Blacksburg, Virginia 24061, USA*

[c] *Macromolecules Innovation Institute and Academy of Integrated Science-Division of Nanoscience, Virginia Tech, Blacksburg, VA 24061, USA*

Corresponding author:

Dr. Wencai Zhang (E-mail: wencaizhang@vt.edu)

The PDF includes:

Figure S1. (a) N_2 adsorption/desorption curve and (b) pore size distribution of PAN fibers.

Figure S2. Atomic concentration (%).

Table S1. Crystal cell parameters of the different Li-PNFs.

Table S2. Weight loss of the different phases for the different Li-PNFs.

Figure S3. (a) PAN and (b) LDH models constructed in DFT calculations.

Table S3. Adsorption kinetic and isotherm fitting parameters of lithium adsorption process using Li-PNFs-1.

Note S1. Kinetics and isotherm fitting models

Table S4. Detailed composition (mg/L) and pH value of the synthetic brine.

Table S5. Experimental parameters.

Figure S4. Photos of the real Li-PNFs fibers.

S1. N_2 adsorption/desorption curve, pore size distribution and atomic concentrations.

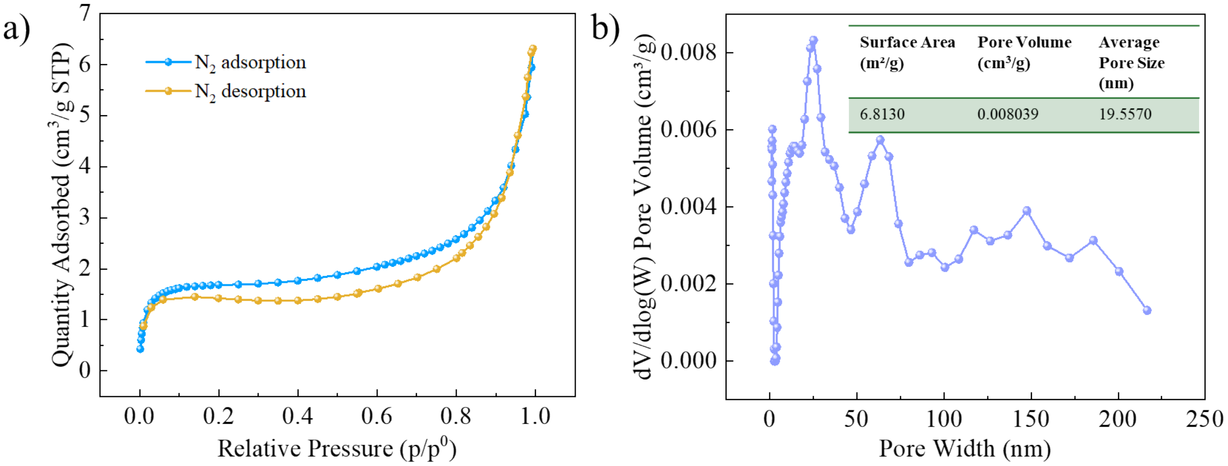

Figure S1. (a) N_2 adsorption/desorption curve and (b) pore size distribution of PAN fibers.

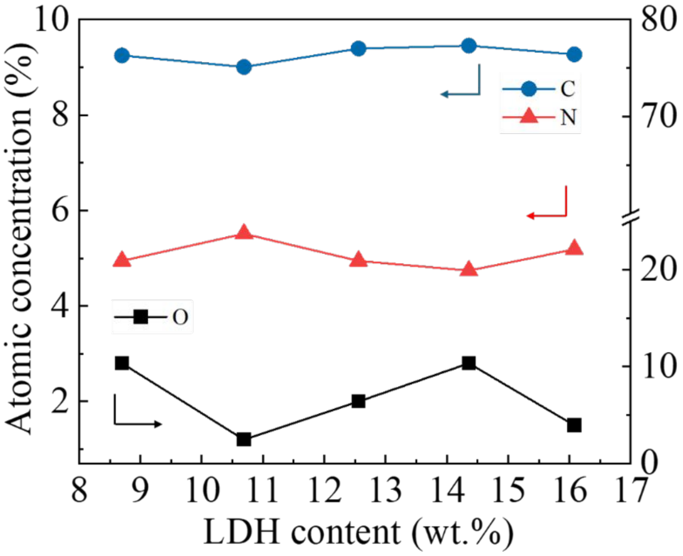

Figure S2. Atomic concentration (%).

S2. Crystal cell parameters and weight loss of the different phases of the different Li-PNFs.

Table S1. Crystal cell parameters of the different Li-PNFs.

	a (Å)	b (Å)	c (Å)	V (Å^3)
Li-PNFs-1	4.98815	4.98815	5.01606	124.81
Li-PNFs-2	5.00391	5.00391	5.00391	125.29
Li-PNFs-3	5.00709	5.00709	5.00727	125.54
Li-PNFs-4	4.99803	4.99803	4.99803	124.85
Li-PNFs-5	4.99444	4.97444	4.99894	125.70

Table S2. Weight loss of the different phases for the different Li-PNFs.

	Weight loss (%)				
	Li-PNFs-1	Li-PNFs-2	Li-PNFs-3	Li-PNFs-4	Li-PNFs-5
Phase I	6.08	5.82	4.39	3.04	6.87
Phase II	5.06	6.49	7.30	7.04	7.15
Phase III	18.59	16.71	18.68	17.36	17.11
Phase IV	43.07	26.76	18.52	40.81	45.74

S3. PAN, LDH models constructed in DFT calculations and adsorption kinetic and isotherm fitting parameters.

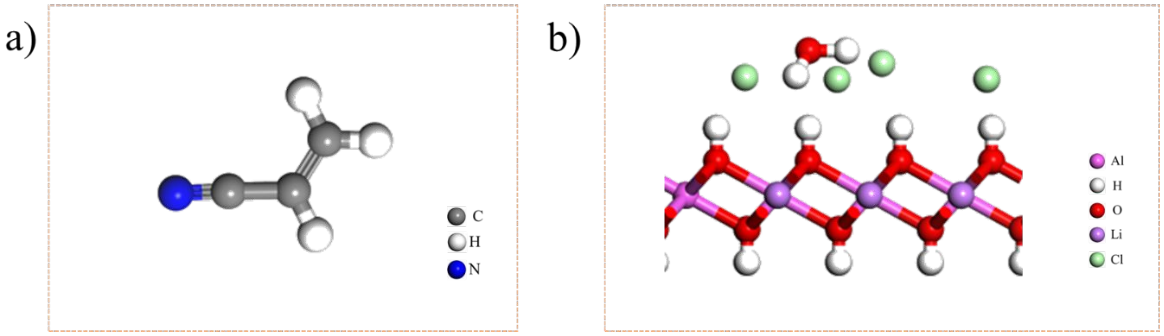

Figure S3. (a) PAN and (b) LDH models constructed in DFT calculations.

Table S3. Adsorption kinetic and isotherm fitting parameters of lithium adsorption process using Li-PNFs-1.

Adsorption kinetics	Q_{max} (mg/g) *	pseudo-first-order model			pseudo-second-order model		
		k_1 (1/min)	q_{e1} (mg/g)	R^2	k_2 (g/(mg·min))	q_{e2} (mg/g)	R^2
	13.0544	0.01356	5.1069	0.6092	0.01392	13.3475	0.9983
Adsorption isotherm	Freundlich	T (K)		K_F (L/g)		R^2	
		293		128.358		0.9328	
	Langmuir	T (K)		q_{max} (mg/g)		b (L/mg)	R^2
		293		117.4401		3.4271·e^{-} 121	0.9887

* Maximum equilibrium adsorption capacity.

S4. Kinetics and isotherm fitting models.

Note S1. Kinetics and isotherm fitting models

Two kinetic models, namely the pseudo-first-order model and pseudo-second-order kinetic model, were applied for curve fitting, with their respective equations provided as $Eq. (S1)$ and $Eq. (S2)$:

$$\ln(q_e - q_t) = lnq_e - k_1 \cdot t \quad \text{(S1)}$$

$$\frac{t}{q_t} = \frac{1}{k_2 \cdot q_e^2} + \frac{1}{q_e} \cdot t \quad \text{(S2)}$$

where q_e (mg/g) means the equilibrium adsorption capacity and q_t (mg/g) means the capacity at time t, k_1 (1/min) and k_2 (g/(mg·min)) are the rate constant.

The Freundlich and Langmuir fitting models, represented by $Eq. (S3)$ and $Eq. (S4)$ respectively, were utilized to analyze the lithium adsorption isotherm.

$$\mathrm{q_e} = \mathrm{K_F C_e^{1/n}} \quad \text{(S3)}$$

where q_e (mg/g) is the equilibrium adsorption capacity and C_e (mg/L) is the equilibrium concentration, K_F refers to the Freundlich isotherm model factor. The n serves as an indicator for both the strength of adsorption and the uniformity of the adsorbent surface.

$$\mathrm{q_e} = \frac{q_{max} b C_e}{1 + b C_e} \quad \text{(S4)}$$

where q_e (mg/g) and C_e (mg/L) have the same meaning as mentioned above. q_{max} (mg/g) is the monolayer adsorption per unit mass of fibers. The constant b in the Langmuir model indicates the affinity between the adsorbate and the adsorbent surface, with higher values of b reflecting stronger adsorption energy.

S5. Detailed composition (mg/L) and pH value of the synthetic brine and experimental parameters.

Table S4. Detailed composition (mg/L) and pH value of the synthetic brine.

Li	Na	Mg	K	Ca	pH value
1,000	120,000	20	25,000	0.32	5.03

Table S5. Experimental parameters.

Sample	DMF (mL)	PAN (g)	LDH powder (g)	Speed (mL/h)	Voltage (kV)	Humidity (%)	Distance (cm)
Li-PNFs-1	20	2	2.0	~4	20	~20	40
Li-PNFs-2	20	2	2.5	~4	20	~20	40
Li-PNFs-3	20	2	3.0	~4	20	~20	40
Li-PNFs-4	20	2	3.5	~4	20	~20	40
Li-PNFs-5	20	2	4.0	~4	20	~20	40

S6. Photos of the real Li-PNFs fibers

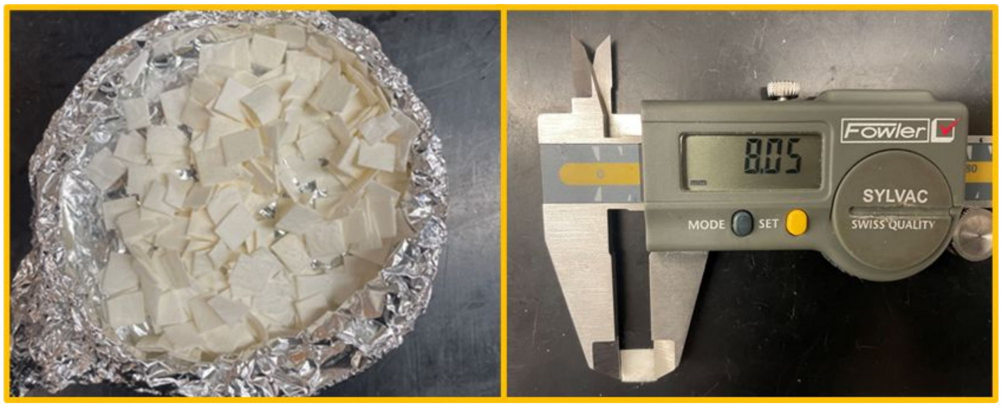

Figure S4. Photos of the real Li-PNFs fibers.
